# Characterization of a Synovial B Cell–Derived Recombinant Monoclonal Antibody Targeting Stromal Calreticulin in the Rheumatoid Joints

**DOI:** 10.4049/jimmunol.1800346

**Published:** 2018-07-25

**Authors:** Elisa Corsiero, Lucas Jagemann, Mauro Perretti, Costantino Pitzalis, Michele Bombardieri

**Affiliations:** * Centre for Experimental Medicine and Rheumatology, William Harvey Research Institute, Barts and The London School of Medicine and Dentistry, Queen Mary University of London, London EC1M 6BQ, United Kingdom; and; † Centre for Biochemical Pharmacology, William Harvey Research Institute, Barts and The London School of Medicine and Dentistry, Queen Mary University of London, London EC1M 6BQ, United Kingdom

## Abstract

Rheumatoid arthritis (RA) is characterized by formation of synovial ectopic lymphoid structures (ELS) supporting B cell autoreactivity toward locally generated citrullinated (cit) antigens, including those contained in neutrophil extracellular traps (NETs). However, only a minority of RA-rmAbs from B cells isolated from ELS^+^ RA tissues react against NETs. Thus, alternative cellular sources of other potential autoantigens targeted by locally differentiated B cells remain undefined. RA fibroblast–like synoviocytes (FLS) have been implicated in the release of RA-associated autoantigens. In this study, we aimed to define stromal-derived autoantigens from RA-FLS targeted by RA-rmAbs. Seventy-one RA-rmAbs were screened toward RA-FLS by living-cell immunofluorescence (IF). Western blotting was used to identify potential autoantigens from RA-FLS protein extracts. Putative candidates were validated using colocalization immunofluorescence confocal microscopy, ELISA, immunoprecipitation assay, and surface plasmon resonance on unmodified/cit proteins. Serum immunoreactivity was tested in anti-citrullinated peptide/protein Abs (ACPA)^+^ versus ACPA^−^ RA patients. Ten out of 71 RA-rmAbs showed clear reactivity toward RA-FLS in immunofluorescence with no binding to NETs. One stromal-reactive RA-rmAb (RA057/11.89.1) decorated a ∼58-kDa band that mass spectrometry and Western blotting with a commercial Ab identified as calreticulin (CRT). Confocal microscopy demonstrated significant cellular colocalization between anti-CRT RA057/11.89.1 in RA-FLS. RA057/11.89.1 was able to immunoprecipitate rCRT. Deimination of CRT to cit-CRT moderately increased RA057/11.89.1 immunoreactivity. cit-CRT displayed increased blocking capacity compared with unmodified CRT in competitive binding assays. Finally, anti–cit-CRT Abs were preferentially detected in ACPA^+^ versus ACPA^−^ RA sera. We identified a synovial B cell–derived RA-rmAb locally differentiated within the ELS^+^ RA synovium reacting toward CRT, a putative novel autoantigen recently described in RA patients, suggesting that FLS-derived CRT may contribute to fuel the local autoimmune response.

## Introduction

Rheumatoid arthritis (RA) is the most common inflammatory erosive polyarthritis, characterized by breach of self-tolerance and production of anti-citrullinated peptide/protein Abs (ACPA). Highly mutated and Ig class-switched ACPA can be manufactured within synovial ectopic lymphoid structures (ELS) displaying features of functional germinal centers (GCs), which develop in around 40% of RA patients (1–3). The frequent observation that hypermutated B cells within ELS in the RA synovium and other autoimmune conditions display evidence of clonal relationship and intratissue clonal diversification supports the current notion that the humoral autoimmune response within ELS, such as those developing in the RA joints, is Ag-driven, leading to the local differentiation of autoreactive B cells (4–8).

Recently, we have shown that synovial RA-rmAbs generated from single CD19^+^ synovial B cells isolated from ELS^+^ ACPA^+^ RA patients recognize locally released citrullinated Ags, such as those contained in neutrophil extracellular traps (NETs) (2). However, anti-NET immunoreactivity only accounts for a minority of the cellular immune reactivity of the large amount of RA-rmAbs that we generated, leading to the hypothesis that alternative cellular sources exist that are responsible for the release of other potential autoantigens targeted by in situ differentiated B cells.

RA fibroblast-like synoviocytes (FLS) play a crucial role in the pathogenesis of RA, directly contributing to local cartilage destruction and synovial inflammation (9–14). RA-FLS are characterized by a sustained, highly proliferative, and activated state with an increased level of antiapoptotic and a decreased level of proapoptotic factors, which induce them to undergo hyperplasia (15, 16). Recently, RA-FLS have been shown to contribute to the local release of citrullinated Ags, particularly in the context of increased autophagy, suggesting that they may contribute to link local inflammation and autoimmunity by acting as an additional source of RA-associated autoantigens (17).

Thus, in this work, we aimed to investigate whether RA-rmAbs generated from single synovial B cells obtained from ELS^+^ ACPA^+^ RA patients display immunoreactivity toward RA-FLS and to identify putative stromal-derived autoantigens fueling the local autoimmune response.

## Materials and Methods

### Patients

Synovial fluids and tissues from RA patients were obtained after informed consent (National Research Ethics Service Committee London: LREC 05/Q0703/198) by aspiration of swollen knees and from total joint replacement, respectively. RA patients were diagnosed according to the revised American College of Rheumatology criteria (Table I) (18).

### Generation of RA-rmAbs from ELS^+^ RA synovial tissue

RA-rmAbs were generated from single synovial CD19^+^ B cells as previously reported (2, 19). Compared to previous work, we obtained 14 additional RA-rmAbs from one additional ELS^+^ ACPA^+^ RA donor, bringing the total of RA-rmAbs tested to 80. Of these, we were able to express 71 RA-rmAbs at sufficient concentration for downstream analysis. Human monoclonal IgG from naive B cells obtained from healthy donors (HD; IgG-2c3) and rmAbs derived from naive and memory B cells from Sjögren syndrome patients were used as controls (19).

### Generation of FLS from RA patients and *sti*mulation of NETosis

FLS were obtained either from synovial tissue or synovial fluid as previously described (11, 20). At 90% confluent, FLS were passaged 1:3 using 0.25% trypsin/EDTA (Sigma). Culture medium was replaced every 3 to 4 d. FLS were used after passage 4 and up to passage 8 to avoid any contamination from synovial macrophages. Neutrophils were isolated from peripheral blood of HD using discontinuous gradient centrifugation and seeded onto cell culture cover slides at 2 × 10^5^ cells per well. Cells were activated with 100 nM PMA for 4 h at 37°C to induce NETosis before fixation with 4% paraformaldehyde (PFA).

### Immunofluorescence microscopy on FLS and NETs

FLS were seeded at 1 × 10^4^ cells per 200 μl onto cover slides. After 24 h, cells were washed in 1× PBS and fixed using either ice-cold 1:1 acetone:methanol or 4% (final concentration) PFA. After washing in TBS and blocking with serum-free protein block (DAKO), RA-rmAbs or control rmAbs were diluted at 50 μg/ml in Ab diluent (DAKO) and applied for 1 h at room temperature (RT). After washing with 1× TBS, Alexa Fluor 488 goat anti-human IgG was applied for 1 h at RT. DAPI (Invitrogen) was added to visualize the nuclei. All sections were visualized using an Olympus BX60 microscope. For double immunofluorescence confocal microscopy in colocalization experiments, RA-rmAbs or control rmAbs were incubated as above. A mouse anti-human calreticulin (CRT) (clone: FMC 75; diluted 1:200; Abcam) was then added for 1 h at RT. After washing, an Alexa Fluor 555–conjugated goat anti-mouse Ab (1:200; Invitrogen) was incubated for 1 h at RT. After washing and mounting, the slides were scanned using a Leica DM5500 confocal microscope. NETs were stained with RA-rmAbs diluted in PBS for 1 h RT. After washing with TBS, Alexa Fluor 488 goat anti-human IgG (1:200; Invitrogen) was added for 30 min at RT. NETs were visualized by DAPI and cit-H4 using a polyclonal rabbit anti-histone H4 (citrulline 3; Millipore).

### Protein extraction and Western blot analysis

All procedures were performed at 4°C using precooled reagents. FLS were washed in ice-cold 1× PBS. radioimmunoprecipitation assay buffer (25 mM Tris-HCl [pH 7.6], 150 mM NaCl, 1% NP-40, 1% sodium deoxycholate, 0.1% SDS) containing Protease Inhibitor Cocktail (Sigma) was added to the cell pellet. After 1 h on ice, sample was centrifuged at 20,000 × *g* for 10 min at 4°C to pellet the cell debris. Supernatant was collected, and the protein concentration was measured using the BCA Protein Assay Kit, following the manufacturer’s instructions (Thermo Fisher Scientific).

Five hundred nanograms of human recombinant CRT (hrCRT) (Abcam) or protein extract obtained from RA-FLS was loaded on 4–20% SDS–polyacrylamide gels (Bio-Rad), and proteins were transferred to a nitrocellulose membrane (GE Healthcare Life Sciences). The blocking was performed in 5% (w/v) nonfat dry milk in 0.1% (v/v) TBST (blocking buffer) overnight at 4°C with gentle agitation, followed by incubation with the primary Ab RA-rmAb or IgG-2c3 at 40 μg/ml in 5% blocking solution for 2 h at RT with agitation. As a positive control for the Western blot, 40 ng of hrCRT was loaded on the gel, and mouse anti-CRT Ab 1:2000 (Abcam) was used for the blotting. After rinsing three times for 10 min in TBST, the membranes were incubated with goat anti-human IgG peroxidase 1:10,000 (Jackson ImmunoResearch Laboratories) or goat anti-mouse IgG peroxidase 1:5000 (Santa Cruz Biotechnology) in 5% blocking buffer for 1 h at RT. The membranes were washed again and incubated for 2 min in Clarity Western ECL substrate (Bio-Rad). Band detection was performed using Hyperfilm ECL (GE Healthcare Life Sciences) and developed in a Konica medical film processor (Konica Minolta). Densitometry analysis was performed using ImageJ software.

### Immunoprecipitation experiment

Immunoprecipitation (IP) was performed by mixing equal amounts (6 μg) of RA-rmAb or IgG-2c3 and hrCRT in 500 μl of Pierce IP Lysis Buffer (Thermo Fisher Scientific) on a rotary shaker for 2 h at 4°C. Protein A Sepharose beads (GE Healthcare Life Sciences) in IP Lysis Buffer were added to the mixture and incubated on a rotary shaker for 2 h at 4°C. After centrifugation and washing three times with cold IP Lysis Buffer, the immunoprecipitates were eluted with 1× Laemmli buffer and resolved using SDS-PAGE. Following the electrophoresis, the gel was stained using SimplyBlue SafeStain (Invitrogen), and the band around 58 kDa was excised and analyzed by mass spectrometry, as described below.

### Enzymatic digestion

In-gel reduction, alkylation, and digestion with trypsin were performed on the excised gel bands prior to subsequent analysis by mass spectrometry. Cysteine residues were reduced with DTT and derivatized by treatment with iodoacetamide to form stable carbamidomethyl derivatives. Trypsin digestion was carried out overnight at RT after initial incubation for 2 h at 37°C.

### Liquid chromatography–tandem mass spectrometry analysis

Peptides were extracted from the gel pieces by a series of acetonitrile and aqueous washes. The extract was pooled with the initial supernatant and lyophilized. The sample was then resuspended in 18 μl of 50 mM ammonium bicarbonate to be analyzed by liquid chromatography–tandem mass spectrometry (LC-MS/MS). Chromatographic separation was performed using an EASY-nLC system (Thermo Fisher Scientific). Peptides were resolved by reversed-phase chromatography on a 75-μm C18 column using a three-step linear gradient of acetonitrile in 0.1% formic acid. The gradient was delivered to elute the peptides at a flow rate of 300 nl/min over 60 min. The eluate was ionized by electrospray ionization using an Orbitrap Velos Pro (Thermo Fisher Scientific) operating under Xcalibur v2.2. The instrument was programmed to acquire in automated data-dependent switching mode, selecting precursor ions based on their intensity for sequencing by collision-induced fragmentation using a Top20 CID method. The tandem mass spectrometry (MS/MS) analyses were conducted using collision energy profiles that were chosen based on the mass-to-charge ratio and the charge state of the peptide.

Raw mass spectrometry data were processed into peak list files using Proteome Discoverer (v1.4; Thermo Fisher Scientific). Processed raw data were searched using the Mascot search algorithm (www.matrixscience.com) against the Uniprot database using All Taxonomy and Human Taxonomy.

### Citrullination of CRT in vitro

hrCRT protein (Abcam) was incubated with rabbit skeletal muscle PAD (7.5 U/mg) in 0.1 M Tris-HCl (pH 7.4), 10 mM CaCl_2_, and 5 mM DTT for 2 h at 50°C. After incubation, CRT was stored at −20°C. Citrullination was confirmed by Western blot analysis using an Anti-Citrulline (Modified) Detection Kit (Merck Millipore) following the manufacturer’s instruction (Supplemental Fig. 1).

### ELISA assay for anti-CRT and inhibition assay

ELISA plates were coated overnight with unmodified/citrullinated CRT protein in 1× PBS at 1 μg/ml. RA-rmAbs or serum samples were transferred into ELISA plates and incubated for 1 h at RT. Unbound samples were removed before incubation for 1 h with HRP-coupled goat anti-human IgG (1:5000). Assays were developed using tetramethylbenzidine (TMB) Substrate Reagent Set (Becton Dickinson Optical Enzyme ImmunoAssay [BD OptEIA]). ODs were measured at 450 nm. All the RA-rmAbs and controls were tested at 50 μg/ml followed by 1:5 serial dilution (CRT protein only). Serum samples were tested after a 1:100 dilution. For the inhibition assay, RA057/11.89.1 Ab at different dilutions was preincubated with unmodified/citrullinated CRT protein at 0.1 μg/ml for 1 h at RT before being transferred to the unmodified/citrullinated CRT-coated plates. Thereafter, ELISA was carried out as described above. Results on RA sera were expressed as arbitrary units (AU). AU = (100/*N*) × OD_450nm_ serum sample, where *N* is the lowest OD_450nm_ value in the anti-CRT Ab in the ACPA^−^ RA patient group.

### Surface plasmon resonance analysis via Biacore platform

All experiments were performed using a Biacore T200 instrument from GE Healthcare Life Sciences. Sensor chip Protein A, designated to bind human Abs, and running buffer 10× HBS-EP+ were purchased from GE Healthcare Life Sciences. Running buffer was diluted 10 times with deionized water, filtered (0.22 μm), and degassed. RA-rmAb or control rmAb was immobilized on the sensor chip surface at 2 μg/ml for 30 s at a flow rate of 10 μl/min. CRT protein diluted at 500, 250, and 125 nM in 1× running buffer was injected for 30 s at a flow rate of 10 μl/min. Running buffer was then flushed for 45 s at a flow rate of 10 μl/min, and finally the chip was regenerated by injecting a glycine solution (10 mM, pH 1.5) for 30 s at a flow rate of 10 μl/min.

### Statistical analysis

Differences in quantitative variables were analyzed by unpaired (two-sample) *t* test and one-way ANOVA (multiple groups) using GraphPad Prism 5.01 software. Correlations were determined using the “rcorr” function in R’s c, which computes a matrix of the Pearson *r* and *p* correlation coefficients for all possible pairs of columns between two input matrices (i.e., anti–cit-CRT Abs and each clinical data point). Missing values were deleted in pairs. A *p* value <0.05 was considered statistically significant. Immunofluorescence colocalization analysis was performed by the Pearson correlation coefficient using ImageJ software (21).

## Results

### A subset of RA-rmAbs derived from synovial B cell clones target RA synovial FLS

**FIGURE 1.**
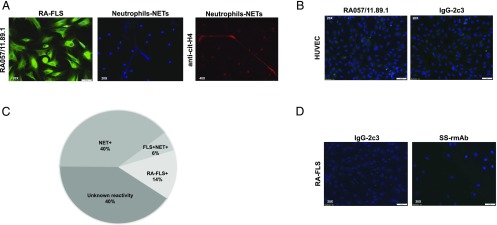
Synovial RA-rmAbs display immunoreactivity toward FLS. (**A**) Representative immunofluorescence picture of RA-FLS and NETs incubated with different RA-rmAbs demonstrating selective immunoreactivity toward FLS-derived Ags (green). NETs were stained by DAPI (blue) and cit-H4 (red) using a polyclonal rabbit anti-histone H4 (citrulline 3; Millipore). (**B**) Representative immunofluorescence pictures of HUVECs incubated with the RA057/11.89.1 rmAb and the control rmAb IgG-2c3. (**C**) Pie chart summarizing the RA-rmAbs’ reactivity toward RA-FLS (14%), FLS-NETs (6%), NETs only (40%), and unknown reactivity (40%). (**D**) Representative immunofluorescence pictures of RA-FLS incubated with control rmAbs, including IgG-2c3 and rmAbs from Sjögren syndrome patients.

Seventy-one RA-rmAbs generated from single synovial B cells (2) were tested for their reactivity toward synovial RA-FLS to assess whether the synovial B cell clones could target stromal-derived autoantigens. Immunofluorescence analysis showed that 10 out of 71 (14%) rmAbs were uniquely reactive toward RA-FLS (Fig. 1A–C), suggesting that anti-FLS and anti-NET Abs are produced by largely independent populations of synovial B cells (Fig. 1B). Conversely, none of the control rmAbs showed reactivity toward RA-FLS (Fig. 1D). As depicted in representative images in Fig. 1A, RA-rmAbs displayed a prevalent anticytoplasmic pattern in RA-FLS, with invariably absent antinuclear immunoreactivity. As shown in Supplemental Fig. 2, the RA-rmAb RA057/11.89.1 conserved the immunostaining on RA-FLS also in nonpermeabilized RA-FLS, suggesting that this Ab can also recognize the cell-surface form of CRT.

### Identification of CRT as an antigenic target of a specific RA-rmAb

**FIGURE 2.**
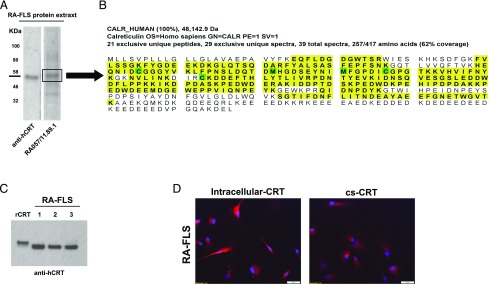
CRT expression in RA-FLS. (**A**) RA-FLS protein extract was subjected to Western blotting and probed with anti-human CRT mAb or RA-rmAb. A protein of around 58 kDa is bound by the RA-rmAb RA057/11.89.1 and anti-CRT Ab. (**B**) Following mass spectrometry analysis by collision-induced dissociation and database searching, peptide and protein assignment detected a high amount of sequence coverage (62%) across the full length of the CRT protein in the RA-FLS protein extract. Twenty-one unique peptides were assigned from a total of 39 tandem mass spectra (MS/MS; highlighted in yellow). Modifications to particular amino acids are highlighted in green. (**C**) Representative CRT expression in RA-FLS from different donors (*n* = 3) by Western blot. (**D**) Representative immunofluorescence pictures of RA-FLS showing expression of intracellular CRT and cell-surface (cs)-CRT (red). For intracellular CRT, RA-FLS were fixed in ice-cold 1:1 acetone:methanol. For cs-CRT, RA-FLS were fixed in 4% PFA. Nuclei were stained with DAPI (blue).

To characterize the stromal autoantigens recognized by the RA-rmAbs, protein extract from RA-FLS was separated on SDS-PAGE, transferred on nitrocellulose membranes, and probed with the RA-rmAbs. As shown in Fig. 2A, one RA-rmAb (RA057/11.89.1) clearly displayed a strong reactivity toward a protein migrating in the ∼58-kDa region. The band at 58 kDa was excised from the gel followed by trypsin digestion into peptides before mass spectrometry analysis (Fig. 2B). LC-MS/MS analysis on the excised band corresponding to the 58 kDa molecular mass confirmed the presence of human CRT, showing that CRT was the third most-represented protein (from over 100 detected by the LC-MS/MS analysis) with a high amount of sequence coverage (62%) across the full length of CRT.

**Table I.** Clinical data of the RA patients used for anti–cit-CRT Abs ELISA assay

RA patients (*n* = 84)	
Gender, %	
Female	72.6
Male	27.4
Age	52.5 ± 1.7
ESR	38.6 ± 3.4
CRP	18.7 ± 3.5
VAS	64.9 ± 2.8
Tender joints	10.3 ± 0.7
Swollen joints	6.7 ± 0.6
DAS28	5.5 ± 0.2
CCP (or ACPA) Abs	65 (ACPA^+^)/19 (ACPA^−^)

The values are expressed as mean ± SEM.

CCP, cyclic citrullinated peptide; CRP, C-reactive protein.

**Table II.** V(D)J gene usage and somatic mutation analysis of RA057/11.89.1

RA057/11.89.1 IgM							
H chain	V_H_	D	J_H_	(−)	CDR3 (Amino Acid)	(+)	Length
	1–18	2–2	6	1	RYCSSTSCYKGSYYYYYYYMDV	2	22
L chain	Vκ		Jκ	(−)	CDR3 (Amino Acid)	(+)	Length
	3–20		4	0	QQYGSSPLT	0	9
Mutations	V Region Nb of Mutations	FR1 Nb of Mutations	CDR1 Nb of Mutations	FR2 Nb of Mutations	CDR2 Nb of Mutations	FR3 Nb of Mutations	CDR3 Nb of Mutations
H chain	1	1	0	0	0	0	0
L chain	12	12	0	0	0	0	0

(−)/(+), negative/positive charges; FR, framework region; Nb, number.

CRT is a conserved chaperone protein mostly expressed in the endoplasmic reticulum that migrates to a ∼58-kDa position in SDS-PAGE (22). Besides MS analysis, we screened in silico for putative targets with similar expected molecular mass in SDS-PAGE based on the proteome analysis of RA-FLS performed by Dasuri et al. (23). Interestingly, CRT emerged as one of the putative matches. Hence, a commercial mouse anti-CRT mAb specifically recognized a band of overlapping molecular mass in RA-FLS protein extracts (Fig. 2A).

We next confirmed the expression of CRT in RA-FLS from different donors by protein immunoassay and cell-based immunofluorescence. As shown in Fig. 2C, CRT was found abundantly in RA-FLS protein extracts. We used hrCRT to confirm the specific binding of the commercial anti-CRT Ab used in Western blot, although hrCRT displayed a slightly higher molecular mass compared with naturally occurring CRT in RA-FLS, probably because of posttranslation modifications in *Escherichia coli*. Using immunofluorescence with a commercial anti-CRT Ab on living cells in permeabilizing and nonpermeabilizing conditions, we demonstrated that CRT can be expressed by RA-FLS both intracellularly and on the cell surface (Fig. 2D).

### The RA-rmAb RA057/11.89.1 targets FLS-derived CRT

**FIGURE 3.**
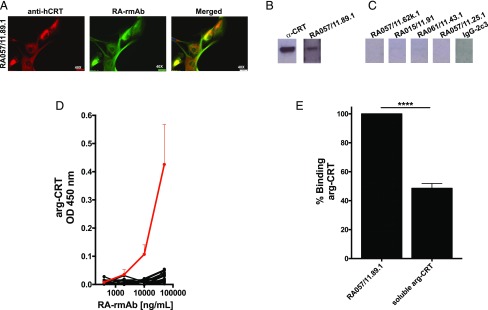
A specific RA-rmAb recognizes unmodified CRT. (**A**) Representative immunofluorescence picture showing staining for CRT (red) and RA-rmAb (green). Nuclei were stained with DAPI (blue). (**B** and **C**) RA-rmAbs binding to arg-CRT in Western blot. As negative control, an rmAb (IgG-2c3) from HD naive B cells was used. (**D**) RA057/11.89.1 RA-rmAb (red line) and negative RA-rmAb binders (black lines) binding to arg-CRT by ELISA. All RA-rmAbs were tested at a concentration of 50 μg/ml followed by four serial dilutions (1:5). Results are expressed as absorbance at 450 nm. The data are the results of two independent experiments. (**E**) Binding inhibition of RA057/11.89.1 RA-rmAb to arg-CRT preincubated with or without soluble arg-CRT (inhibitor). Results are expressed as percentage of binding inhibition. The data are the results of three independent experiments. ****p < 0.0001.

We then confirmed that the RA-rmAb RA057/11.89.1 specifically targets FLS-derived CRT. As shown in Fig. 3A, double immunofluorescence staining with RA057/11.89.1 in combination with an anti-CRT Ab in permeabilizing conditions, analyzed with confocal microscopy, demonstrated a strong cellular colocalization with CRT and the RA-rmAb, which also recognizes CRT in RA-FLS protein extracts. The degree of colocalization between the two fluorophores was quantified using ImageJ, with the Pearson correlation coefficient showing a strong correlation (*r* = 0.92). We next used hrCRT to screen the RA057/11.89.1 Ab by Western blot. As shown in Fig. 3B, this RA Ab confirmed the binding toward hrCRT in Western blot, whereas not only RA-rmAbs with no binding to RA-FLS in cell-based immune screening but also other RA-FLS–reactive RA-rmAbs failed to recognize CRT in Western blot (Fig. 3C, Supplemental Fig. 3, respectively).

The binding toward arg-hrCRT was quantitatively assessed by screening the RA057/11.89.1 in ELISA. The RA-rmAb showed binding to CRT in a dose-dependent manner (Fig. 3D, red line). In contrast, a large majority of RA-rmAbs failed to display any binding to CRT (Fig. 3D, black lines). We used inhibition assay to further confirm whether CRT protein was recognized by the RA-rmAb. As shown in Fig. 3E, preincubation of the RA057/11.89.1 Ab with CRT reduced the binding to arg-CRT protein to around 60%.

**FIGURE 4.**
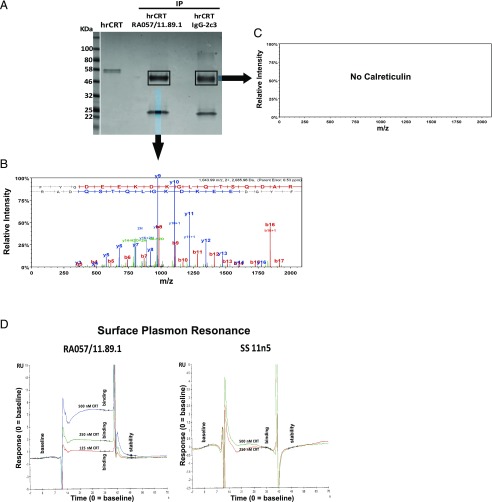
Binding studies of RA057/11.89.1 toward CRT. (**A**) IP of hrCRT and RA057/11.89.1 or IgG-2c3. As control, hrCRT alone was loaded. Coomassie staining is shown. (**B** and **C**) CRT protein was detected in the IP sample RA-rmAb/CRT following LC-MS/MS and database searching against the human portion of the Uniprot database but not in the IP sample with the control rmAb IgG-2c3. Example of a fragmentation spectra of a peptide from the CRT protein detected in the IP sample is shown. MS/MS fragmentation spectra of a peptide ion with mass-to-charge (m/z) ratio of 1043.99^2+^. Fragment ions annotated with the y-series are database assigned from the C-terminal end of the peptide, whereas fragmentation peaks annotated with the b-series arise from the N-terminal end of the peptide. A strong consecutive matching of the peaks in both series provides strong evidence for the correct database assignment of the spectra to the peptide in the protein of interest. (**D**) Sensorgrams showing binding of RA057/11.89.1 RA-rmAb and one control rmAb to CRT protein used at different concentrations. Binding is expressed as responsive unit (*y*-axis) over time (*x*-axis).

To corroborate the CRT/RA-rmAb binding results, IP assays were performed. As shown in Fig. 4A, IP of hrCRT with the RA-rmAb displayed a band around 58 kDa. Although we observed a similar band using the control rmAb IgG-2c3, because the H chain of the Igs (50 kDa) migrates in the same region of CRT (data not shown), LC-MS/MS analysis of the excised immunoprecipitate complexes clearly identified CRT in the IP CRT-RA057/11.89.1 sample but not in the IP CRT-IgG2c3 sample (Fig. 4B, 4C). Finally, binding to CRT was confirmed by surface plasmon resonance via Biacore platform (Fig. 4D).

### RA-rmAb binding characterization toward deiminated CRT

**FIGURE 5.**
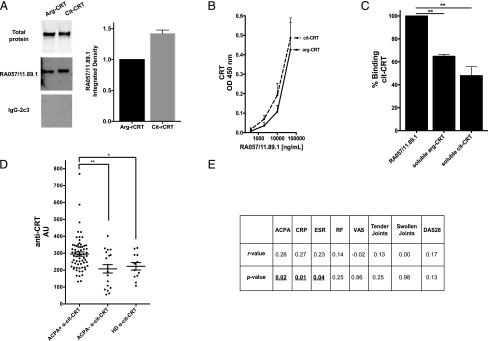
RA057/11.89.1 immunoreactivity toward deiminated CRT and expression of anti–cit-CRT Abs in serum of RA patients. (**A**) Left panel, RA057/11.89.1 RA-rmAb was tested in Western blot toward arg-CRT versus cit-CRT. Total arg-/cit-CRT protein is shown in the top blot. Right panel, Densitometry analysis of the Western blot is shown. Data were normalized toward total protein for arg-CRT and cit-CRT, respectively. (**B**) RA057/11.89.1 RA-rmAb binding to arg- and cit-CRT by ELISA. RA-rmAb was tested at a concentration of 50 μg/ml followed by four serial dilutions (1:5). Results are expressed as absorbance at 450 nm. (**C**) Binding inhibition of RA057/11.89.1 RA-rmAb to cit-CRT preincubated with or without soluble arg- or cit-CRT (inhibitor). Results are expressed as percentage of binding inhibition. (**D**) Anti–arg-CRT and anti–cit-CRT Ab level in serum from ACPA^+^ RA patients (*n* = 65), ACPA^−^ RA patients (*n* = 19), and HD (*n* = 16) measured by ELISA. Results are expressed as AU. AU = (100/*N*) × OD_450nm_ serum sample, where *N* is the lowest OD_450nm_ value in the anti–arg-CRT Ab in ACPA^−^ RA patient group. (**E**) Summary table showing correlation of serum anti-cit-CRT Abs with ACPA, CRP, ESR, RF, VAS, tender/swollen joints, and DAS28 score. The data in (A), (B), and (D) are the results of two independent experiments, whereas data in (C) are the results of three independent experiments. *p < 0.05, **p < 0.01. CRP, C-reactive protein.

We next investigated whether the identified RA-rmAb with anti-CRT immunoreactivity displayed enhanced binding toward an in vitro citrullinated form of CRT (cit-CRT). CRT’s primary structure has eight arginine residues that are potential sites of citrullination. Thus, unmodified CRT (arg-CRT) was deiminated in vitro by peptidyl arginine deiminase 2 (PAD2), and citrullination was confirmed by Western blotting using a specific anti-citrulline Ab (Supplemental Fig. 1). We used both Western blot and ELISA to screen the RA-rmAb toward unmodified and deiminated CRT. As shown in Fig. 5A, densitometry analysis of SDS-PAGE Western blot suggested that the anti-CRT RA-rmAb displayed an increased binding toward cit-CRT. Similar data were observed in ELISA toward citrullinated versus arg-CRT, as depicted in Fig. 5B. As represented in Fig. 5C, preincubation of the RA-rmAb with deiminated CRT induced a greater decrease (around 60%) in the binding to in vitro cit-CRT compared with preincubation with soluble arg-CRT.

### Increased levels of anti–cit-CRT Abs in sera from ACPA^+^ RA patients

We finally investigated the prevalence of anti-CRT Abs in a cohort of 84 patients with early arthritis who were naive to any treatment and part of the Pathobiology of Early Arthritis Cohort (http://www.peac-mrc.mds.qmul.ac.uk/), and 16 HD. Serum anti–cit-CRT Abs were measured by ELISA using deiminated hrCRT (Fig. 5D, Supplemental Fig. 4). Early RA patients were divided into ACPA^+^ (*n* = 65) and ACPA^−^ (*n* = 19) based on conventional anti-CCP2 test, with HD serum samples used as controls. As shown in Fig. 5D, Ab levels to cit-CRT in ACPA^+^ RA patients were significantly increased compared with ACPA^−^. Analysis was performed to evaluate correlation between anti–cit-CRT Abs in RA patient sera and the levels of ACPA, CRP, erythrocyte sedimentation rate (ESR), rheumatoid factor (RF), visual analog scale of pain (VAS), tender/swollen joints, and disease activity score (DAS) 28 score at baseline (Fig. 5E). A significant correlation was observed between anti–cit-CRT Abs, ACPA (*r* = 0.26, *p* = 0.02), CRP (*r* = 0.27, *p* = 0.01), and ESR (*r* = 0.23, *p* = 0.04).

## Discussion

The identification of ELS developing in the joints of RA patients as functional sites of B cell affinity maturation and the evidence that within ectopic GCs B cells undergo intrasynovial clonal diversification strongly indicated that humoral immune responses in the RA synovium are driven by locally released (auto)antigens (4–6, 8).

To investigate the cellular sources and the nature of the Ags recognized by hypermutated synovial B cells, we optimized a method to generate full rmAbs from B cells single-sorted from ELS^+^ synovial tissues from ACPA^+^ RA patients. So far, we have generated over 80 RA-rmAbs that display for the vast majority highly mutated Ig H and L chain V genes and evidence of intratissue affinity maturation (2). In previous work, we identified a subset of around 40% of RA synovial B cells derived from ectopic GCs that displayed reactivity toward Ags released by NETs, and we characterized these autoantigens as primarily citrullinated histones H2A and H2B (2). In the present work, we explored the possibility that alternative, non-NET cellular sources exist in the RA joints that are capable of releasing other potential autoantigens, driving the local adaptive immune response in the RA synovial tissue. In particular, we focused our attention on FLS, a key proinflammatory component of the RA synovitis that contain a high amount of putative RA-associated autoantigens in their deiminated form, such as vimentin and α-enolase, as shown in a proteomic profiling of RA-FLS (23). Additionally, recent work that we contributed to demonstrated that the induction of autophagy in RA-FLS favors the generation of citrullinated Ags, suggesting that RA-FLS may contribute to inflammation and autoimmunity also by releasing RA-associated autoantigens in the synovial microenvironment (17).

Therefore, we initially screened our RA-rmAbs using indirect immunofluorescence with live primary RA-FLS from different donors as substrate. By this method, we identified 10 (14%) RA-rmAbs with clear immunoreactivity to RA-FLS without any binding to NETs. Of relevance, all the anti-FLS clones displayed a prevalent cytoplasmic pattern in immunofluorescent staining and for the vast majority were immunoreactive using both permeabilizing and nonpermeabilizing methods.

Using immunoblot from RA-FLS protein extracts, we observed that one RA mAb (RA057/11.89.1) was strongly reactive toward a ∼58-kDa band. Analysis of the V(D)J gene usage for both H and L chains revealed that RA057/11.89.1 mAb was characterized by V_H_1-18/D2-2/J_H_6 and Vκ3-20/Jκ4 gene segments. Furthermore, the original isotype of this clone was Igμ, which sustained the low number of mutations observed in the H chain V region (*n* = 1). Instead, we observed a higher number of mutations in the L chain V region (*n* = 12). A detailed analysis of this clone is reported in Table II.

Mass spectrometry of RA-FLS protein extract, in silico analysis of RA-FLS proteomic profiles, and a series of coimmunoblot and colocalization confocal microscopy experiments identified the 58-kDa band as CRT. CRT is a conserved chaperone protein that migrates into the 58-kDa position in SDS-PAGE (22), is mainly expressed in the endoplasmic reticulum, and is responsible for Ca^2+^ transportation and folding of glycoproteins (24). CRT can also be expressed on the cell surface, playing a critical role in the clearance of apoptotic cells (25), and can be released in the extracellular environment via the secretory pathway (26). It is formed by three domains: 1) N-terminal domain, 2) middle domain (named P-domain), and 3) C-terminal domain. CRT has been found to be abundantly expressed in RA-FLS (23), and several studies indicated a higher concentration of CRT in the serum and synovial fluid of RA patients compared with osteoarthritis and HD serum samples that correlated with RA disease activity (27, 28). Increased levels of CRT in the synovial tissue of RA compared with osteoarthritis patients have been also demonstrated (24, 25, 27–29). Interestingly, it has been shown that CRT recognizes the RA shared epitope HLA domain sequence and can modulate the signaling activated by the shared epitope ligand when present in its citrullinated form (29). Moreover, although native CRT has been described as an autoantigen in several autoimmune conditions (30, 31), its role as a target of autoreactive B cells in RA has only very recently been investigated, with the demonstration that around 60% of RA patients display circulating anti–cit-CRT Abs (32).

In our work, we first confirmed, to our knowledge, that CRT was highly expressed in RA-FLS not only intracellularly but also on the cell surface using a highly monoclonal anti-CRT Ab. The specific reactivity of one of our RA-rmAbs (RA057/11.89.1) with CRT was then confirmed by using at least three methods: 1) colocalization with anti-CRT in confocal microscopy; 2) Western blot using RA-FLS protein extracts and/or hrCRT as substrates and IP using CRT and the RA-rmAb, followed by LC-MS/MS analysis; and 3) ELISA using hrCRT with competitive binding assays. We also generated cit-CRT by deiminating CRT with PAD2 in vitro and demonstrated using both immunoblot and ELISA that the anti-CRT rmAb identified displayed enhanced binding to the citrullinated compared with the native form of CRT, with preincubation with cit-CRT able to decrease RA057/11.89.1 immunoreactivity by 60%.

To confirm the results obtained at the single synovial B cell clonal level with the systemic autoantibody production in RA patients, we tested the reactivity of 84 patients with early RA toward cit-CRT. We significantly detected anti–cit-CRT Abs more frequently in the serum of ACPA^+^ RA patients compared with those with a negative ACPA status, expanding on recent data obtained in established RA patients (32) and suggesting that CRT acts as an autoantigen already in early stages of RA in a subset of patients. Interestingly, anti–cit-CRT Abs were significantly and positively correlated with ACPA, CRP, and ESR levels, although their clinical significance in the context of the ACPA family remains to be elucidated in larger prospective cohorts.

Nevertheless, our work highlights CRT as a novel autoantigen locally released in the RA synovial compartment that appears to promote local humoral autoimmunity. These data are also of interest in line with recent studies showing that cell-surface CRT in its citrullinated form can enhance the binding to a shared epitope ligand and can activate downstream innate and adaptive immune cell signaling, which is in keeping with the notion that ACPA are strongly associated with the shared epitope amino acid sequence of the HLA-DR β-chain (shared epitope) (25, 29, 33–35). Whether anti-CRT Abs could interfere with the strength of signaling from the proposed CRT–shared epitope complex remains to be formally elucidated, but it has been proposed that these Abs might affect the binding of cit-CRT to the shared epitope ligand, thus influencing the inflammatory cascade activated by this interaction (32). Likewise, further experiments are needed to investigate whether anti-CRT Abs can modulate RA-FLS function and promote a proinflammatory phenotype in these cells. The characterization of RA057/11.89.1 as an anti-CRT rmAb provided in our work will pave the way for such functional experiments.

In summary, in this work we identified synovial B cell clones diversified within RA synovial ELS that react against RA-FLS–derived autoantigens, and we characterized RA057/11.89.1 as a novel mAb targeting stromal-derived CRT. These results, linked with recent data reporting a high prevalence of anti-CRT Abs in RA patients, suggest that CRT can act as a locally released autoantigen that can be targeted by autoreactive B cells.

## Supplementary Material

Data Supplement
