# Supplementary material for: Characterization of a Synovial B Cell–Derived Recombinant Monoclonal Antibody Targeting Stromal Calreticulin in the Rheumatoid Joints
Source: J Immunol. 2018 Jul 25;201(5):1373–81. doi: 10.4049/jimmunol.1800346 (PMC6099528; doi:10.4049/jimmunol.1800346)
Supplement: Data Supplement [file JI_1800346.zip › JI_1800346_Supplemental_Figures_1.pdf]

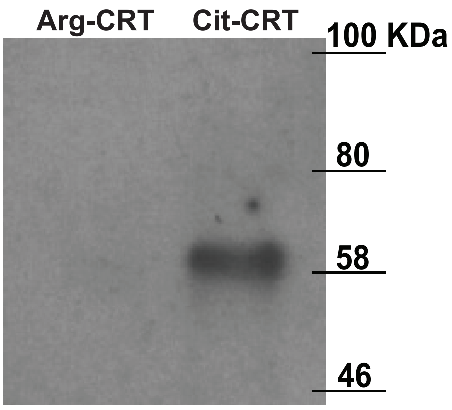

Supplementary figure S1: ***In vitro*** **citrullination of CRT by PAD enzyme.**

Western blot using an anti-citrulline (modified) detection kit showing *in vitro* citrullination of hrCRT by PAD enzyme. Left lane: arginine-containing CRT. Right lane: citrullinated-containing CRT. CRT molecular weight ~ 58-KDa.

For Peer Review. Do not distribute. Destroy after use.

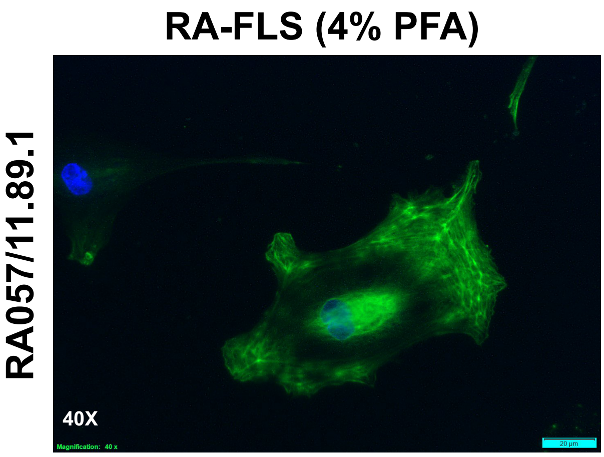

Supplementary figure S2: RA057/11.89.1 rmAb displays cell-surface staining in RA-FLS. Representative immunofluorescence picture of RA-FLS fixed in 4% paraformaldehyde (PFA) and incubated with the RA-rmAb RA057/11.89.1 (green). Nuclei were stained with DAPI (blue).

For Peer Review. Do not distribute. Destroy after use.

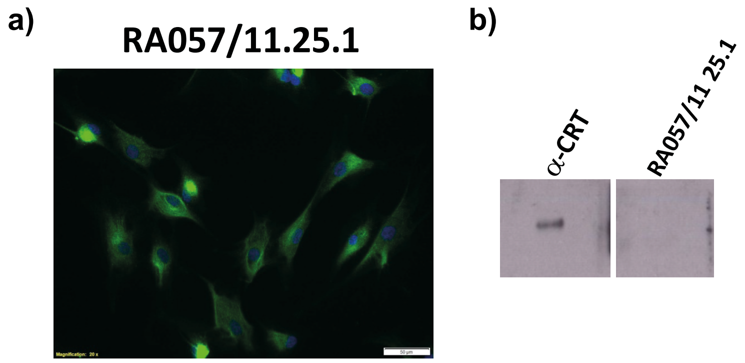

Supplementary figure S3: RA-rmAb binding RA-FLS but not calreticulin (CRT). (a) Representative immunofluorescence picture showing RA-rmAb binding to RA-FLS (green) displaying **(b)** no binding to arg-CRT in Western blot. As control for the Western blot, hrCRT was loaded and mouse anti-CRT antibody was used for the blotting.

For Peer Review. Do not distribute. Destroy after use.

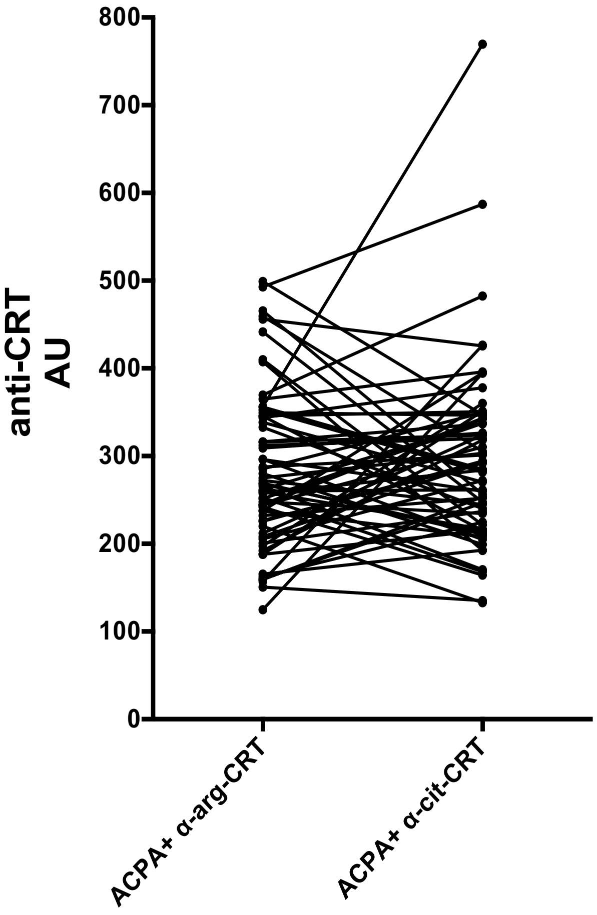

Supplementary figure S4: Anti-CRT antibodies in the serum of RA patients. Comparison of the binding of the patients' sera to unmodified and cit-CRT. Results are expressed as arbitrary units (AU).
